# *TUM-ParticleTyper*: A detection and quantification tool for automated analysis of (Microplastic) particles and fibers

**DOI:** 10.1371/journal.pone.0234766

**Published:** 2020-06-23

**Authors:** Elisabeth von der Esch, Alexander J. Kohles, Philipp M. Anger, Roland Hoppe, Reinhard Niessner, Martin Elsner, Natalia P. Ivleva

**Affiliations:** Institute of Hydrochemistry, Chair of Analytical Chemistry and Water Chemistry, Technical University of Munich, Munich, Germany; University of Montpellier, FRANCE

## Abstract

*TUM-ParticleTyper* is a novel program for the automated detection, quantification and morphological characterization of fragments, including particles and fibers, in images from optical, fluorescence and electron microscopy (SEM). It can be used to automatically select targets for subsequent chemical analysis, e.g., Raman microscopy, or any other single particle identification method. The program was specifically developed and validated for the analysis of microplastic particles on gold coated polycarbonate filters. Our method development was supported by the design of a filter holder that minimizes filter roughness and facilitates enhanced focusing for better images and Raman measurements. The *TUM-ParticleTyper* software is tunable to the user’s specific sample demands and can extract the morphological characteristics of detected objects (coordinates, Feret’s diameter min / max, area and shape). Results are saved in csv-format and contours of detected objects are displayed as an overlay on the original image. Additionally, the program can stitch a set of images to create a full image out of several smaller ones. An additional useful feature is the inclusion of a statistical process to calculate the minimum number of particles that must be chemically identified to be representative of all particles localized on the substrate. The program performance was evaluated on genuine microplastic samples. The *TUM-ParticleTyper* software localizes particles using an adaptive threshold with results comparable to the “gold standard” method (manual localization by an expert) and surpasses the commonly used *Otsu* thresholding by doubling the rate of true positive localizations. This enables the analysis of a statistically significant number of particles on the filter selected by random sampling, measured via single point approach. This extreme reduction in measurement points was validated by comparison to chemical imaging, applying both procedures to the same area at comparable processing times. The single point approach was both faster and more accurate proving the applicability of the presented program.

## Introduction

Microplastic (MP) may be formed from plastic over time by, fragmentations under the influence of UV light and mechanical abrasion, as well as oxidation and biological breakdown [1]. MP has been found in air [2–4], water [3, 5–7] and soil samples [8]. However, MP particles are very challenging to analyze, as the term “microplastic” describes a heterogeneous mixture of polymer types (at varying stages of degradation), sizes (1 μm-1 mm) and shapes (fragments, fibers, films, and spheres). Consequently, chemical and morphological heterogeneity is combined with low analyte concentrations in the respective samples and a high contamination potential from any plastic material used during sampling or processing [9]. Ideally, all chemical and morphological characteristics, such as polymer types, size distribution and number concentration, of MP should be analyzed and quantified for each sample to answer the question: “How many MP particles are in the sample?”

The general scheme for single particle analysis of MP is a workup step for the extraction and purification of MP [10] after which all remaining particles–microplastic as well as residual environmental colloids–are deposited on a smooth filter surface. The smoothness of the filter is of high importance, as any subsequent measurement, be it Fourier-transform infrared spectroscopy (FTIR) or confocal Raman microspectroscopy, will depend on a flat surface to enable optimal focus on the particles [11, 12]. This is especially true if automated routines are used, where particles are first identified by acquiring images for a morphological assessment, including the determination of the particle centers for the subsequent measurement. Programs enabling these automated routines are commercially available and open source alternatives exist [13–18]. However, almost all routines lack a calibration and validation tool. The problem with the validation of a particle localization program is that spheres are typically used to demonstrate segmentation efficiency. This is a valid procedure and has the benefit that a ground truth is easily accessible through computer generated images. Unfortunately, this does not accurately validate the procedure for the multitude of shapes and color inhomogeneities within the sample. Another possible validation procedure is to extract images from several publications, representing several image capture devices and settings, and then analyzing those images with the processing routine in question [15]. This is a good routine to show the generalizability of the implemented functions but lacks the ground truth for each image. A third path is to apply an automatic thresholding routine, which can be overruled by the user to “make the segmentation look good”. This is also a valid approach, used in most commercial software, as the current gold standard for the identification of particles in images is still the human operator. The drawbacks of this approach are the missing reproducibility, its high dependency on the operator and the lack of validation possibilities. Therefore, we focused on building a particle detection program (*TUM-ParticleType*r) that can be calibrated and assembled a manageable validation procedure in accordance with With et al. and Udupa et al. [19, 20]. It can be transferred to the output of the readers preferred software. The initial focus was on microscopy images taken with darkfield illumination, and it was subsequently adapted to the analysis of SEM and fluorescence images.

Merely detecting and morphologically characterizing the particles is not enough as so far, the results produced by any image processing routine do not include the chemical properties of the particles [21]. So, after this first step we are still unable to distinguish between microplastic and native particles and can therefore not yet answer the question: “How many microplastic particles do we have in our sample?” At this stage, results from the particle detection can be used, however, to substantially reduce the measurement time of the sample. By only targeting the particle centers the number of e.g. Raman spectra to be measured and classified via database matching is reduced to the number of particles found in the sample. Provided that the measurement of only one spectrum at the particle’s center is representative for the entire particle, this reduction is common practice [5, 6, 9] and was implemented into *TUM-ParticleTyper*. The reduction was nonetheless tested and validated through a comparison with a chemical image of the same area, analyzed in a comparable time frame. Area and time were chosen as fixed parameters, as the area of the filter, which is measured, is synonymous with representatively in the case of imaging, and time is the variable that needs to be optimized. This means that the resolution and timeframe of the mapping process is set to match the “single point measurement at each particle” strategy.

Even so, assuming that a filter contains only 200 000 particles and one spectrum is acquired in the center of each particle, sample analysis of would still take N (number of particles, i.e. 200 000) * t (acquisition time e.g. 20 s) = 47 days. Thus, a subsampling on the filter is a requirement for feasible MP analysis. There are currently many subsampling schemes [5, 6, 22]. However, it has not been determined, which strategy yields the most accurate extrapolation. A random sampling tool was implemented into the software to allow a sample reduction according to Anger and von der Esch et al. 2018 [9], the csv-output file generated by *TUM-ParticleTyper* can be used to extract the particle coordinates for any selection scheme.

In this project, it was our objective to create a particle detection software that operates on Raman microscopy, fluorescence microscopy and scanning electron microscopy (SEM) images. Furthermore, the goal was to deliver calibration and validation tools for the particle detection within the images. The validation protocol should be generally applicable and transferrable to the output of any other particle detection software. For Raman microspectroscopy, an additional validation addressed the often-used single point measurement approach for chemical characterization. Further, to reduce the overall measurement time, it was our goal to implement a subsampling routine into the software. To show the prospects and limits of our automated morphological and chemical characterization routine, we subsequently applied it to a washing machine water sample.

The paper was split into three parts: 1) The main text, which informs on the general analysis routine and highlights the strengths and challenges of *TUM-ParticleTyper*. 2) The supplementary information, which gives details on the experiments conducted for the development, calibration, and validation of the program. 3) The software documentation, which gives details on the program itself and highlights the functions used for the particle detection. The program documentation, the *TUM-ParticleTyper* software and test images are available freely in our *TUMmedia* repository [23]. This partitioning of the publication was necessary so that each target group can easily find the necessary information for their purpose 1) general information 2) and 3) for the reproduction of our results and for the application of *TUM-ParticleTyper*. Furthermore, it enabled us to write an interactive documentation, where the functions of our program can easily be looked up, while coding.

## Material and methods

### Roughness testing for the development of filter holders

To define a parameter to optimize the smoothness of filters as prerequisite for optimal focus in FTIR or Raman spectroscopy, the flattening potential of different filter fixation techniques was evaluated by measuring the maximum peak-to-peak distance. This is the distance of the highest to the lowest pixel on the surface: the smaller this distance is, the smoother is the surface and the better the fixation method. For details, we refer to the full procedure in the SI section 1.1.

### Production of reference materials for the development of the image processing program and optimization of image acquisition procedures

Reference materials were produced by ultrasonication of solid polymers and filtrated onto gold coated polycarbonate filters [24]. The filters carrying the reference materials were then used to optimize the camera settings of the Raman microscope (*alpha300R* Raman Microscope, WITec GmbH, Germany) and the scanning electron microscope (*Sigma 300 VP*, Carl Zeiss AG, Germany).

The most important parameters when producing images for the characterization of particles are 1) contrast, 2) definition, 3) resolution, and 4) color range of the image. The settings used for Raman microscopy, fluorescence microscopy and SEM can be found in the SI section 1.2.

### Acquisition of chemical information via Raman microspectroscopy

Chemical information can be acquired by measuring Raman spectra and comparting the spectra with a database. For this study, spectra at a single position (particle centers determined by *TUM-ParticleTyper*) were measured as well as maps, which combine many short measurements at specified distances to create a chemical image according to the procedure developed by Käppler et al. 2016 [25]. For details, we refer to the full procedure in the SI section 1.3.

### Fiber detection in washing machine water

To test the applicability of our particle localization and characterization program, a washing machine sample was deposited on a filter and processed with *TUM-ParticleTyper*. The goal was to determine how many textile microfibers were present in the sample and to characterize them chemically via Raman spectroscopy. For details, we refer to the full procedure in the SI section 1.4.

## Results and discussion

### Morphological and chemical analysis of MP reference materials via *TUM-ParticleTyper*

#### Flat filter surfaces as a prerequisite for optimal focus

A smooth surface is a prerequisite for optimal focus in a confocal measurement with Raman microspectroscopy [9]. One possibility to achieve this is to use inherently stiff filter materials like silicon wafers [12]. However, silicon wafers are expensive and show a very strong Raman signal, which may interfere with the identification of MP. Therefore, a subtraction of the silicon signal from all spectra before a database matching is required. Alternative filter materials were tested by Ossmann et al. 2017 [11], who found aluminum-coated polycarbonate filters to have the lowest interference in the recorded particle spectra. Furthermore, they found that the particles are best visualized using darkfield illumination delivering highly defined and high contrast images of particles down to 1 μm [11]. As alternative gold coated polycarbonate filters can be used for Raman and infrared spectroscopy [6, 26]. To combine both a smooth surface and low signal interference, a series of filter holders was developed (S1 Fig in S1 File) and tested with commercially available gold-coated polycarbonate filters. After optimization the roughness, which was expressed as the distance of the highest to the lowest part of the filter on 12 mm × 12 mm area could be reduced from originally 63.1 μm to 5.8 μm, which is comparable to a silicon wafer (details in SI section 2.1).

#### Localization and morphological characterization of particles with *TUM-ParticleTyper*

**Fig 1:**
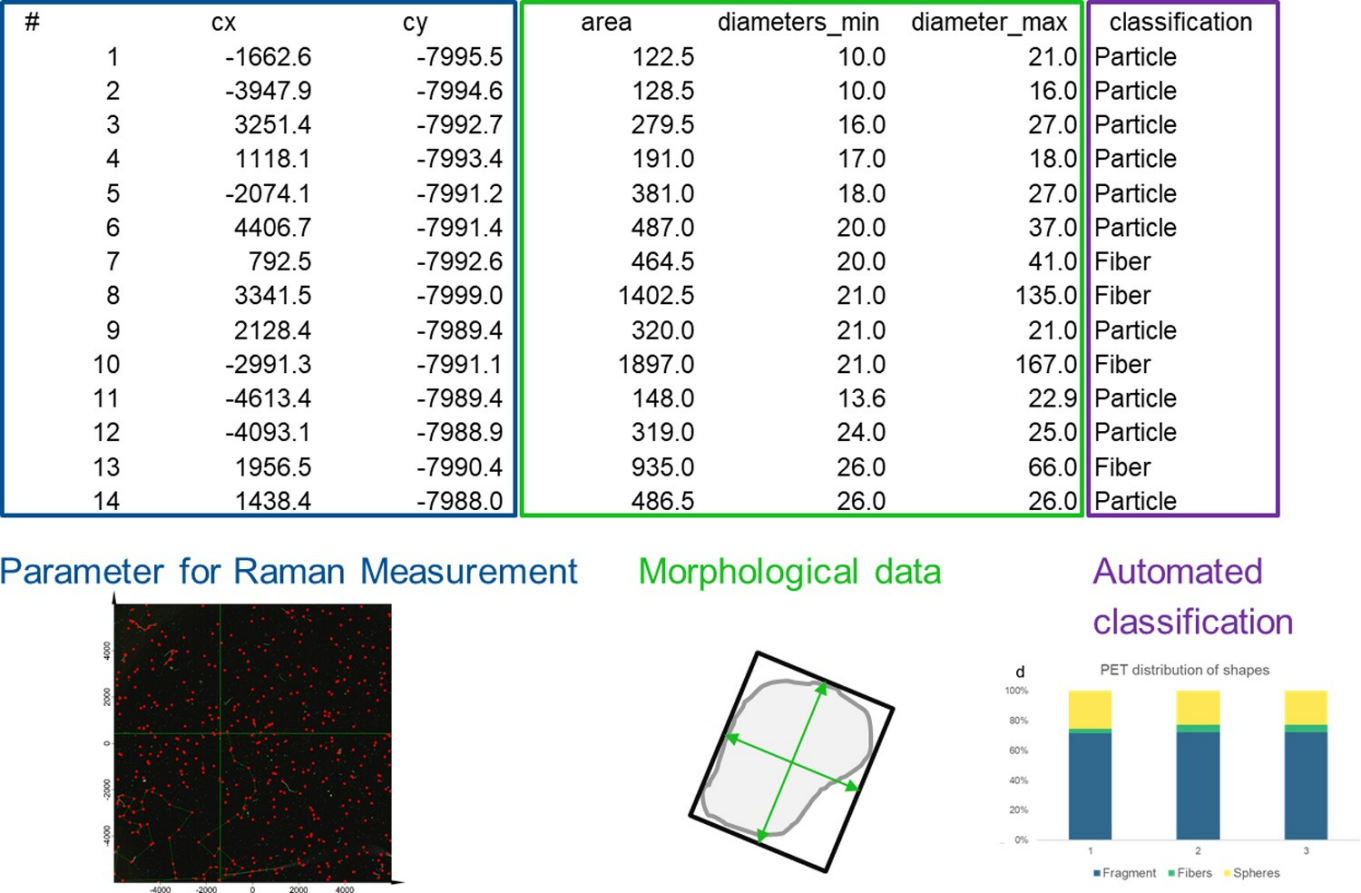
*TUM-ParticleTyper* output. Parameters needed for a subsequent Raman measurement (blue box); morphological data (green box) represented by the area (pixel that exceed the brightness threshold within the determined contour); diameters min / max (determined by the Feret’s method, meaning that the width and length of the smallest possible box that encloses the contour yield the diameters); the classification in particles and fibers (purple box).

Independent of the original image (fluorescence, optical, or SEM), the *TUM-ParticleTyper* delivers three outputs. First, an overlay of the original image with the extracted contours is created. Therefore, the user can see what particles were recognized and roughly assess the success of the automatic particle detection. This is not to be confused with a proper validation. Second, a black and white image with all detected particles is generated. This can be used to transfer the particle detection information into any other software using an automatic thresholding technique, as only black and white pixels exist. For example, the user can combine the *TUM-ParticleTyper* localization with the automated measurement of the *Witec ParticleScout* or any software that allows the import of images and the assignment of a space transformation. Third, should a graphical input not be possible the *TUM-ParticleTyper* delivers a csv-file that contains the measurement coordinates and the morphological features of the detected particles (Fig 1). If the system (fluorescence microscope, Raman microscope, FTIR microscope or SEM) has a way of importing coordinates via csv, this is how coordinates can be assigned for subsequent Raman, FTIR, or EDX measurements. We decided to create this set of outputs rather than trying to control any measurement devices directly. This has the benefit that if the measurement device can load images or csv files the particle locations can be transferred to it.

**Fig 2:**
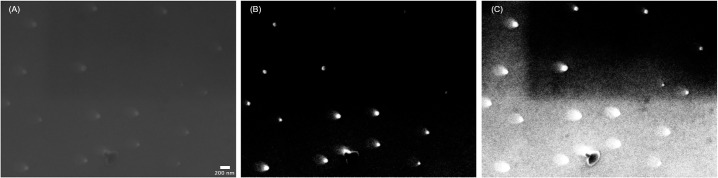
When image enhancement fails. Original SEM image of PS spheres of nominally d = 80 nm (A); SEM image with slightly enhanced contrast and brightness (B); SEM image with strongly enhanced contrast and brightness (C).

The challenge for any particle detection software is to automatically identify the contours of all particles and fibers depicted in the image that match the user’s input specifications. To this end, the image is first transformed into gray scale. Thereafter often a global thresholding method (like *Otsu* [15, 27]) is applied to the image. This might lead to different results in different parts of the image if the lighting or the background is inhomogeneous. In addition, since not all particles share the same gray values (some appear darker, some appear lighter), global thresholding will not result in optimal outcome. Even though increasing contrast and brightness could separate the image strictly into black and white so that the use of a global threshold could work in theory, the parametrization is difficult in practice. Enhancing the contrast and brightness too much also increases the noise in the image, which is then detected as particles leading to artefacts. Furthermore, the hard-coded parameters are not very well generalizable and different settings, in which the optical image was taken, might lead to different qualities of particle detection. This issue is illustrated by Anger and Prechtl et al. 2019 [15], who presented an open source software package based on *Otsu’s* algorithm, which was built to enable detection and morphological characterization of particles. A common workaround for this problem is to implement features into the program where the user can adjust contrast, brightness or the threshold itself. However, as Prata et al. 2019 pointed out this approach, while sometimes effective, leads to non-reproducible results. An example image that challenges these adjustments is the following SEM image (Fig 2).

**Fig 3:**
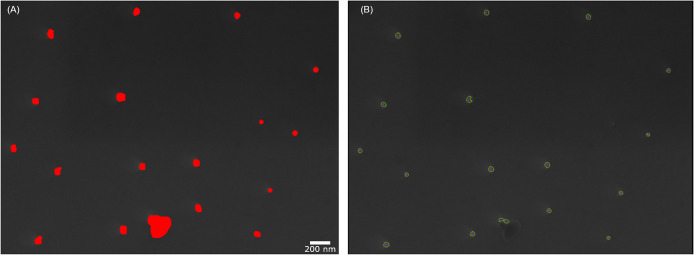
Successful particle detection with *TUM-ParticleTyper*. Sample SEM image with marked ground truth in red (A) and SEM image analyzed by *TUM-ParticleTyper* (B).

As can be seen in Fig 2 the particles at the bottom of the image become better visible when increasing the contrast and the brightness of the image slightly, whereas the particles at the top are still not recognizable. Only when enhancing them further do they become apparent, but at the price of a greater noise in the lower half of the image. This is the reason why a global-thresholding fails for these kinds of images. The logical consequence is to change the thresholding approach to suit the variable lighting conditions and the brightness range of the particles. Therefore, the global thresholding strategy was changed to an adaptive threshold, where only pixels in proximity influence the threshold in the *TUM-ParticleTyper* software. Our procedure also starts with the transformation of the image into a gray scale image. Subsequently the contours are found by using an adaptive threshold with a Gaussian window [28]. The background interference problem is alleviated by blurring the image and then applying the adaptive threshold. The blurring is required to reduce the runtime of the particle detection and to prevent random noise from being falsely detected as particles. With this procedure, we were able to solve the problem, as can be seen in Fig 3 (Further information on the program sequence can be viewed in the program documentation [23]).

Another challenge was that the area of the particles and the size of the Gaussian window influenced each other. A large Gaussian window resulted in excellent detection of large particles, but poor recognition of small particles. When the Gaussian window was small, the opposite effect was observed. For a more accurate detection, the image is analyzed twice, first with a large window to find the large particles only. The second run with the small Gaussian window focuses on the small objects only. This two-step process leads to 5 trainable hyperparameters: The neighborhood size and C-value (constant subtracted from the mean of the neighborhood) of each of the two thresholds and the size boundary between small and large objects. Their parametrization is described later. An overview is given in Table 1:

**Table 1:** Trainable hyperparameter for the adaptive threshold.

Name	Meaning	Effect	Typical Range
**Neighbourhood Size**	The number of nearby pixels considered for the thresholding of each pixel.	Higher number of pixels: More accurate detection of larger particles. Worse detection of smaller particles.	> 49 pixels for a large window
**small / large**			> 9 pixels for a small window
		Smaller number of pixels: vice versa.	
			Scales with the resolution of the image
**C-value**	A constant subtracted from the weighted mean of the neighbourhood pixels	Larger constant: smaller difference between brightness of object and brightness of background needed for a detection.	[-10; +10]
**small / large**			
		Smaller constant: vice versa.	
**Size boundary**	Decision boundary for which particles will be considered during the first run of the program and which in the second.	Higher number of pixels: Used when we expect the particles to be larger.	> 50 pixels
			Scales with the resolution of the image.
		Smaller number of pixels: Used when we expect the particles to be smaller.	

**Fig 4:**
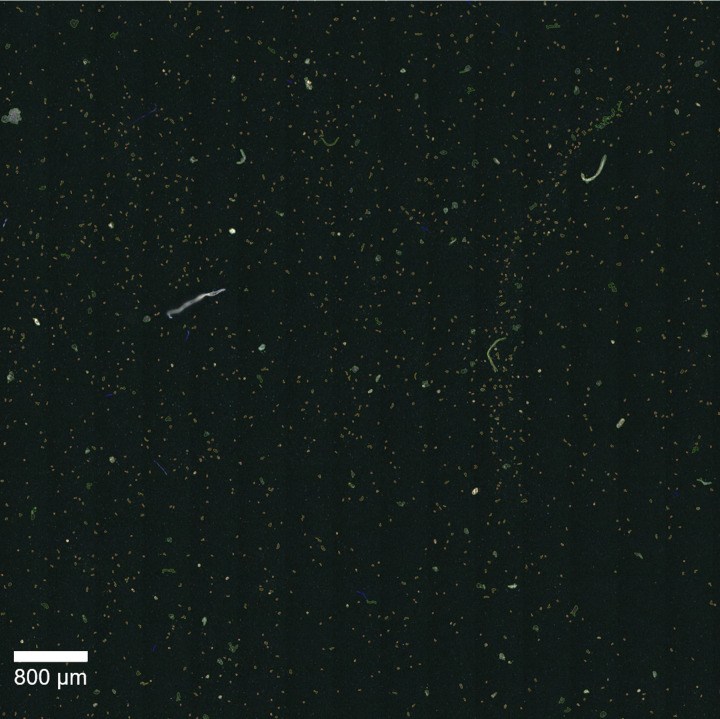
Sample optical image for Raman analyzed by *TUM-ParticleTyper*. Full scale images can be downloaded from our TUMmedia repository [23]. The scale bar was added after processing, as it is otherwise recognized as an object by the image processing software.

The program generates and saves the input image in gray values, with contours of particles marked in green (particles detected by the first run with the large gaussian window) or yellow (particles detected by the second run small gaussian window) and contours of fibers marked in blue. Additionally, their centers are depicted. The analyzed example image can be seen in Fig 4.

**Fig 5:**
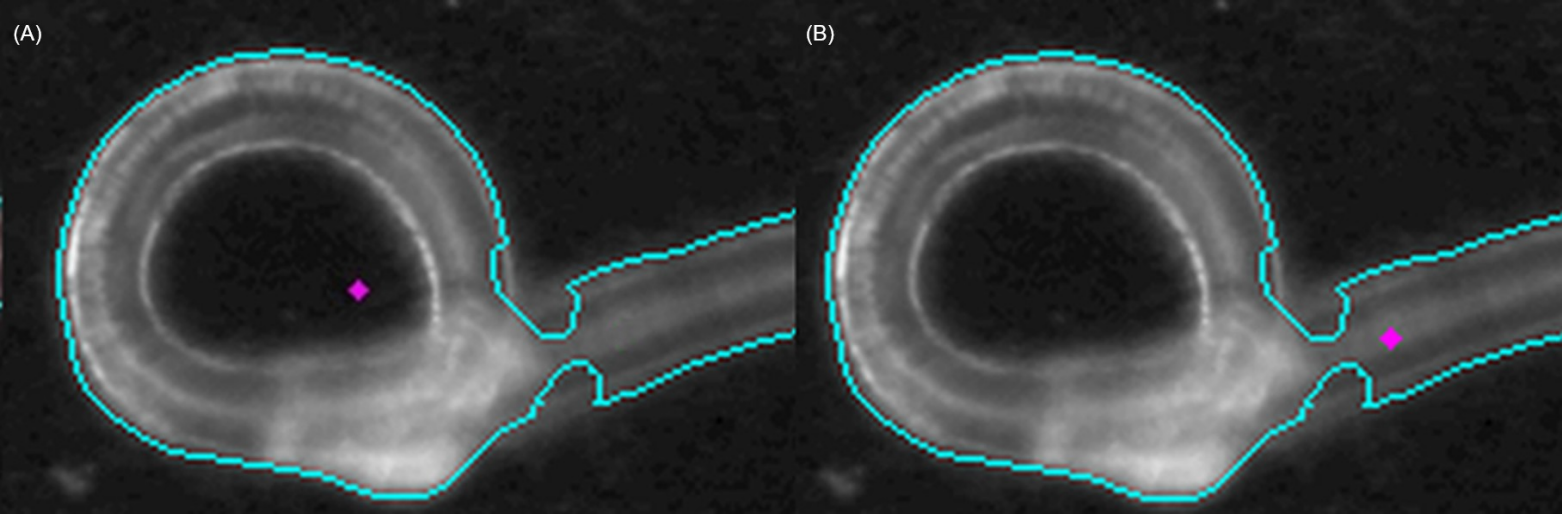
Center correction for curved fiber. The contour is drawn in turquoise. Originally calculated center (A). Corrected center (B).

After all contours were extracted, the morphological characteristics for each particle could be calculated. For further characterization, the area, coordinates of the center, and Feret’s diameters are required, as these yield the size distribution of the sample and enable us to classify the particle shape roughly in general categories (particle or fiber). This estimation was done by checking the ratio between the maximum and the minimum Feret's diameter to be larger than 2.0 and checking the ratio between the product of minimum and maximum Feret's diameter and the area of the contour to be larger than 4.0. If either of these criteria is fulfilled, the object will be classified as a fiber, otherwise the object will be classified as a particle (further information in the documentation under challenges in the analysis). The centers of the particles are used for a subsequent Raman measurement in our case. One problem is however, that the center of a contour does not always lie within the contour (e.g. when the contour is bow-shaped) or lies inside of a hole within the contour (e.g. when the object is torus-shaped). An example can be seen in the following image (Fig 5).

These cases can be detected firstly by checking the color of the pixel on which the center is positioned (a dark pixel indicates the background) and secondly by calculating the distance to the contour, which will be negative if the center lies outside of the contour and positive otherwise. Therefore, centers within holes, centers outside of contours or centers not far enough on the inside of a contour, will be drawn far inside of the contour by the program. This ensures that the laser does not miss the object or hits it just on its edge. Therefore, yielding a robust coordinate selection for the measurement of the particle’s or fiber’s spectrum.

**Fig 6:**
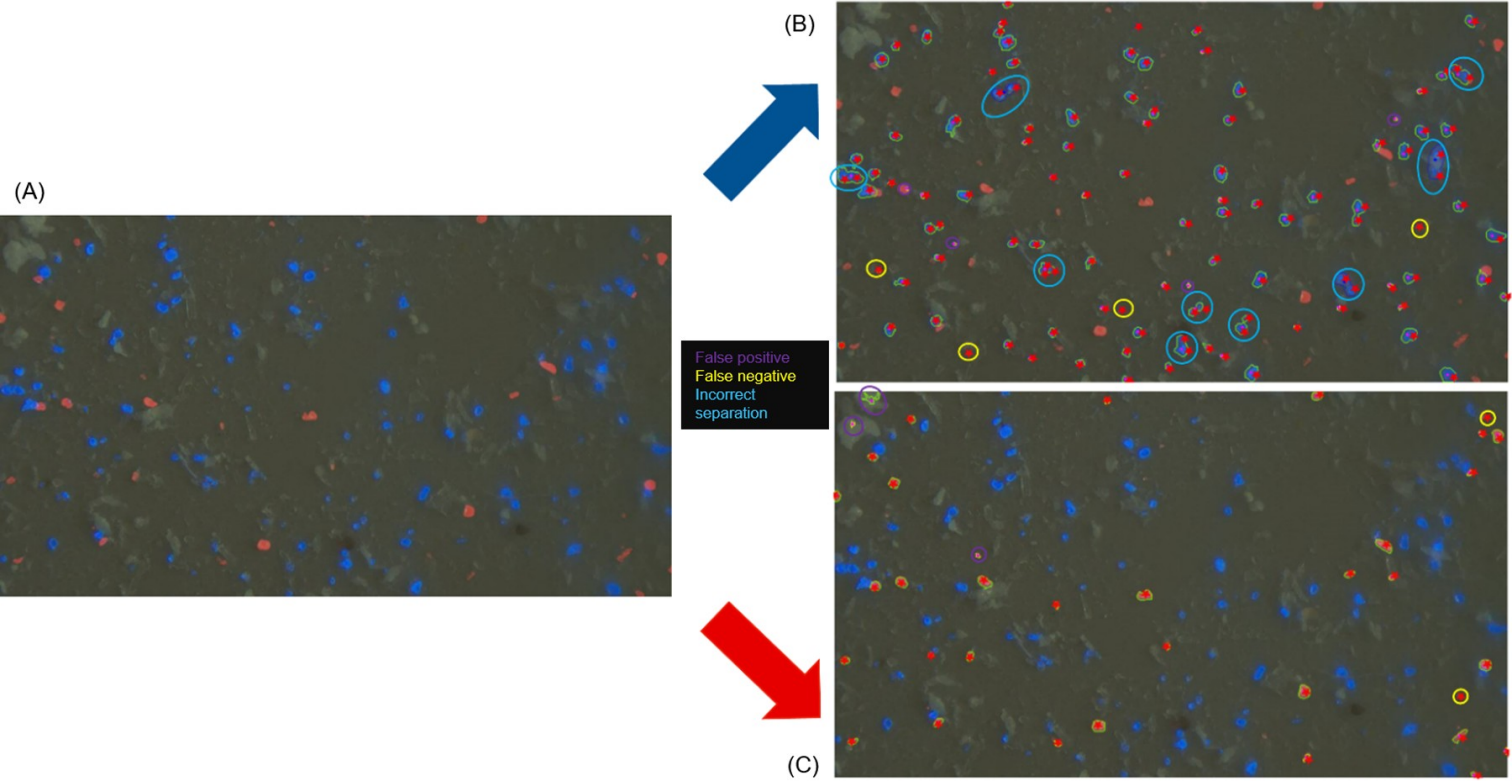
Particle localization using the fluorescence channels. Original fluorescence microscopy image by Hannes Imhof and Astrid Bartonitz, TUM, Aquatic Systems Biology Unit (A). *TUM-ParticleTyper* output for the blue channel (B) and for the red channel (C). Additional processed images can be found in the repository [23]. False positives are marked in purple, false negatives in yellow and incorrect separation is marked in blue. The expert particle assignment is indicated trough red stars. The evaluation is further described in Table 2.

Another challenge was the separation of particles that lie in proximity to each other or are agglomerated. An example of such situations is depicted in the following fluorescence image (Fig 6). The task was to selectively detect and quantify the dyed MP fragments, ideally by their respective color.

**Table 2:** Evaluation of the particle localization using the fluorescence channels.

	Operator	*TUM-ParticleTyper*	False positive	False negative	Incorrect separation
**Blue channel**	106	96	4	4	20 in 9 inst.
**Red channel**	33	34	3	2	0

The filtering of colors is achieved through a preprocessing step. The analysis mode is used to select the RGB-color, all other color values are set to zero. Therefore, only particles with the fitting colors are distinguished from the black background. After the selection, the contrast and brightness are enhanced to ensure that the particles appear white after the transformation into gray values. For the fluorescence images automatic thresholding via *Otsu* was found to be suitable, so it was applied here while Raman and SEM images require the Gaussian window thresholding. Both the blue and the red stained particles can be detected separately and a comparison between operator and *TUM-ParticleTyper* shows, that the program delivers reasonable results. In the processed images we see that the borders of the particles fit the contours of the particles very well and result in good size estimates. We could however not completely overcome the problem that grouped or agglomerated particles are detected as a large particle. In this instance 20 particles were detected as 9 particles, leading to a lower particle count by the *TUM-ParticleTyper*. Overall, we observe that the particle counts are similar but if we investigate the objects detected as particles, we do see differences between the operator and the software. Which is why not only the total particle number but also the false positives and false negatives should be considered during validation. As red and blue selective channels were already introduced into the software, a green selective channel was added to enable the preselection of microplastic through Nile red staining for subsequent Raman identification. Nile Red is a fluorescent dye that has been used to stain both pristine and aged microplastic [16, 29, 30]. While Shim et al. 2016 reported false positive and false negative staining of microplastic in the range of 100 μm – 300 μm, Erni-Cassola et al. 2017 found that all microplastics in the range of 20 μm – 1000 μm were stained by this dye (n = 60 overall, n_polymer_ = 37, negative control n_nonpolymer_ = 23). This makes fluorescent preselection for further IR or Raman analysis an attractive way to reduce the number of particles that need to be analyzed, if and only if, the method indeed provides an effective staining on environmental microplastic.

An alternative to reduce the sample size, by random sampling was also implemented. This feature selects an appropriately large subset of measurement targets according to the users specifications on the margin of error, confidence interval and estimated microplastic content of the sample [9]. The selection will then be exported into a separate file.

### Parametrization

A very important part of this project is the parametrization and validation of the program. To make sure that the program’s output is as close as possible to a predefined consensus value, it is necessary to tune the hyperparameters accordingly and to make sure that the program’s error be as small as possible. To perform this parametrization, it was separated into different steps:

1. Creating the consensus value: To evaluate the program's performance and to adapt hyperparameters based on an error function, a consensus value is needed. Therefore, an expert manually analyzed seven images for each Raman and SEM and marked all particles and fibers in red. Using the "red_fluorescence" function of the program, it is easily possible to extract all needed information for each particle from the labeled images. The most important information hereby is the number and position of the particles.
2. Performing grid-search: A search over all five hyperparameters (neighborhood size small / large, C-value small / large and the size boundary) is performed. All combinations of hyperparameters within a certain range are tested using a predefined step size. A smaller step size results in a longer runtime of the search but ensures a thorough search. The result is the test of all possible combination of hyperparameters within the defined range. This range was not chosen arbitrarily but based on the experience of prior analysis using the program and in a way that analyses with useless results are omitted. Besides the program's usual output, the summed up area, summed up minimum *Feret's* diameters and the summed up maximum *Feret's* diameters of all particles of each image are saved.
3. Evaluating the grid-search results: After the grid-search, the best-performing hyperparameter sets are selected. This is done by comparing the mean of the relative errors of the number of found particles, the total size of all found particles and the sum of each particle's minimum and maximum Feret's diameter for each image. The mean of these four relative errors is used as a comparative value between all iterations of the grid search. A smaller value indicates a more accurate result.
4. Investigating the best results: The results of the elected hyperparameter sets are manually checked regarding the following classifications.
  - True Positive: An object of the consensus value was detected at the same position as one object in the analysis.
  - False Negative: An object of the consensus value was not detected.
  - False Positive: An object was detected in the analysis with no corresponding object of the consensus value.

**Fig 7:**
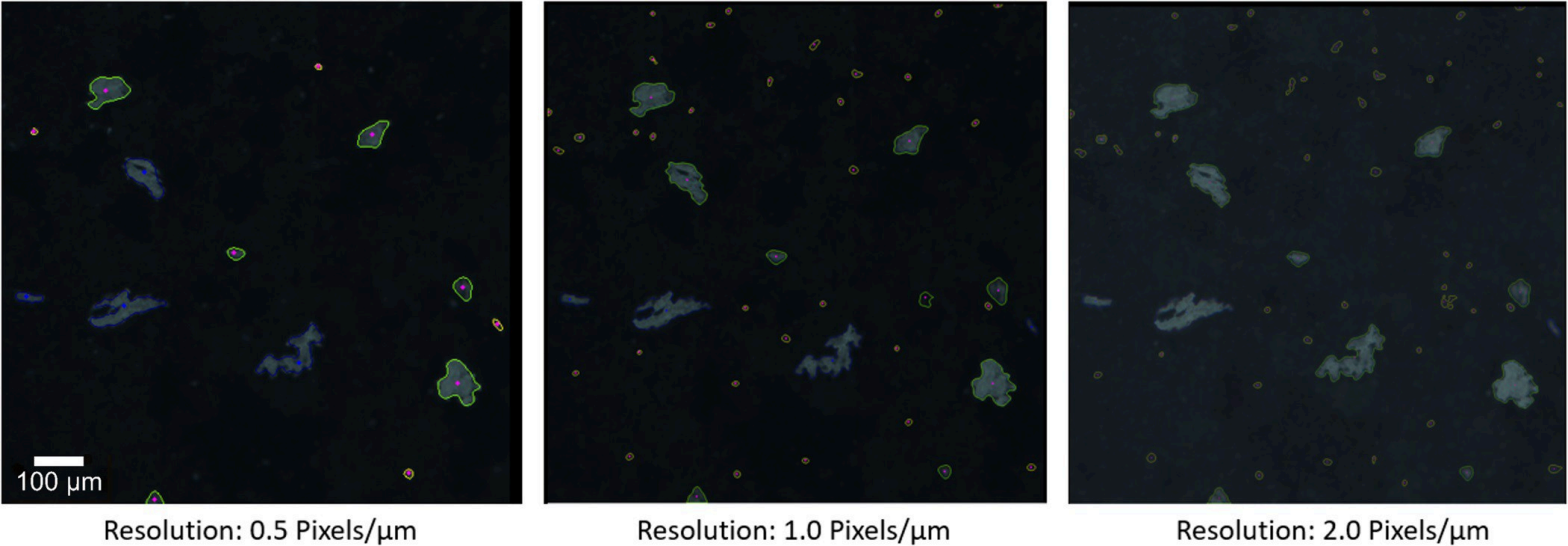
Results of the analysis of an image with different resolutions.

Based on these steps the best performing hyperparameter set (neighborhood size small / large, C-value small / large and the size boundary) was chosen and implemented in the software. Since images of different use cases might vary, these values can be adjusted in the program code to fit the application. Additionally, the neighborhood sizes of the adaptive threshold scale with the resolution of the image, since the same particle occupies a different number of pixels in a low-resolution image and a high-resolution image, which means that also a different number of neighborhood pixels must be considered for the same result. Oftentimes the image’s quality has a larger impact on the analysis than small variations within the parameters. As can be seen in Fig 7, with decreasing number of pixels to represent an image (hence decreasing resolution), the information contained in the image decreases and therefore the number of detectable particles decreases too, and the shapes of the detected objects are less detailed.

Furthermore, it is important to mention that there is no "perfect" parametrization. There are a lot of parameter sets that work well on the images and produce similar results. But dependent on the image, the objects and the task, different parameters might lead to better results. During the evaluation of the particle assignment of two experts, we found that they produce deviant results, which is in accordance with Prata et al. 2019 [13]. Leading to the conclusion there is a margin in uncertainty / inaccuracy that can be tolerated when analyzing the optical images.

**Fig 8:**
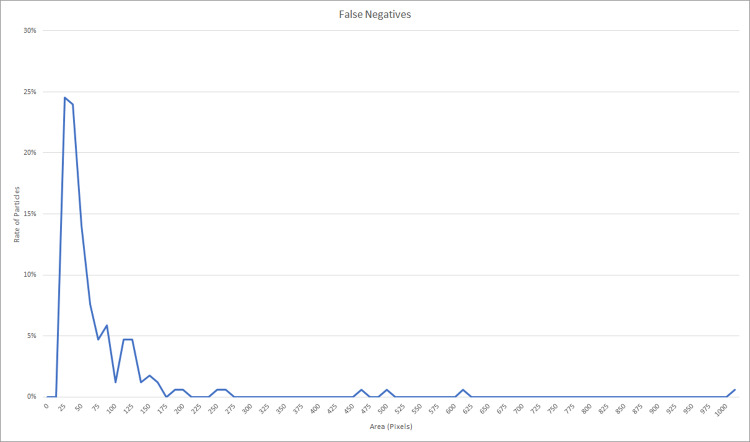
Size distribution of particles non detected with *TUM-ParticleTyper* during parametrization of optical images for Raman.

Example: For the first grid-search based on optical images for Raman, the results for the constraints we searched with (minPixels of 20, min Feret's diameter of 5 and resolution of 0.5 pixel/ μm) resulted in 171 false negatives (particles that were not detected) in all test images and showed that 62.6% occurred for object areas below 51 pixels, when using the best performing parametrization. The limit of a minimal area of 51 pixels for the successful detection is the limiting factor for the lowest detectable particle area but this area is relative to the resolution. Therefore, if smaller particles are to be detected a higher resolution is necessary. With our current setup we are limited to particles larger than 10 μm (Fig 8). By taking smaller images with the same objective we can distribute the maximal number of pixels (8000 × 8000) on a smaller area (e.g. 4000 μm × 4000 μm) creating a higher resolution (resolution = 2 pixel/ μm) image enabling the search for smaller particles.

This implies that the *TUM-ParticleTyper* is limited regarding small particles. When reanalyzing the parametrization considering only objects larger than 50 pixels, the parametrization used above was still in the top 5 of best-performing parameters and the number of false positives decreased substantially. The other four top results had very similar parameters.

### Validation

For the validation of a program for image analysis and to ensure its functionality it is recommended to test its performance before the application. The performance was validated according to the following six factors for "performance evaluation in image processing" [19]:

1. Accuracy: How well has the algorithm performed with respect to some reference?
  The accuracy is covered during the parametrization step, when the program's performance is compared to the consensus value of the expert.
2. Robustness: An algorithm’s capacity for tolerating various conditions.
  With the use of an adaptive threshold the algorithm can overcome inhomogeneous conditions in images (e.g. lighting). To test the algorithm’s robustness, a real-life sample from a washing machine was analyzed. Even though the filter is overloaded with particles and dried foam, which built a cake on top of the filter, the *TUM-ParticleTyper* was able to detect fibers on this cluttered surface.
3. Sensitivity: How responsive is the algorithm to small changes in features?
  In general, the adaptive threshold works independent of the shape of the particle. It's size, however, is the most influential feature on the detection quality. When the user chooses the “minPixels” input-value too small, the algorithm might detect a high number of false positives and false negatives. It is therefore very sensitive to decreasing sizes of particles. However, this can be overcome by capturing high resolution images possibly also switching to higher magnification objectives and choosing values for “minPixels” accordingly.
4. Adaptability: How well does the algorithm deal with variability in images?
  The adaptability of the algorithm is demonstrated by the different modes (Raman, SEM and flourescence) it can handle. Additionally, the program was tested for proper functioning not only with images taken in our own laboratory, but also with images from other publications and therefore from other cameras and camera setups. Since promising results were achieved, the program's ability to adapt to different images has been demonstrated.
5. Reliability: The degree to which an algorithm, when repeated using the same stable data, yields the same result.
  Since the algorithm is deterministic, every analysis of an image using the same parameters results in the same found contours. Additionally, tests with flipped, rotated and cropped images were performed. They all generated the same results. Deviations only occurred at the edges of the cropped images since objects were cut-off and therefore the area or diameters did not fit anymore.
6. Efficiency: The practical viability of an algorithm.
  Since the program needs to handle large-size images, blurring the image before the extraction of contours ensures that small particles (noise) will be reduced or removed. This is important to guarantee an acceptable runtime. Since the algorithm focuses on particles starting at a certain size, the neglection of smaller ones is not a problem. To show the enormous improvement regarding the time of analysis, a comparison between the expert’s time on creating the consensus value and the program’s runtime was made on the test images for SEM. While the expert needed approximately 16 seconds to find and mark a particle, the program requires approximately 1 millisecond for each particle (on the developer’s machine. Results may vary). This results in a speedup of a factor over 1500.

**Fig 9:**
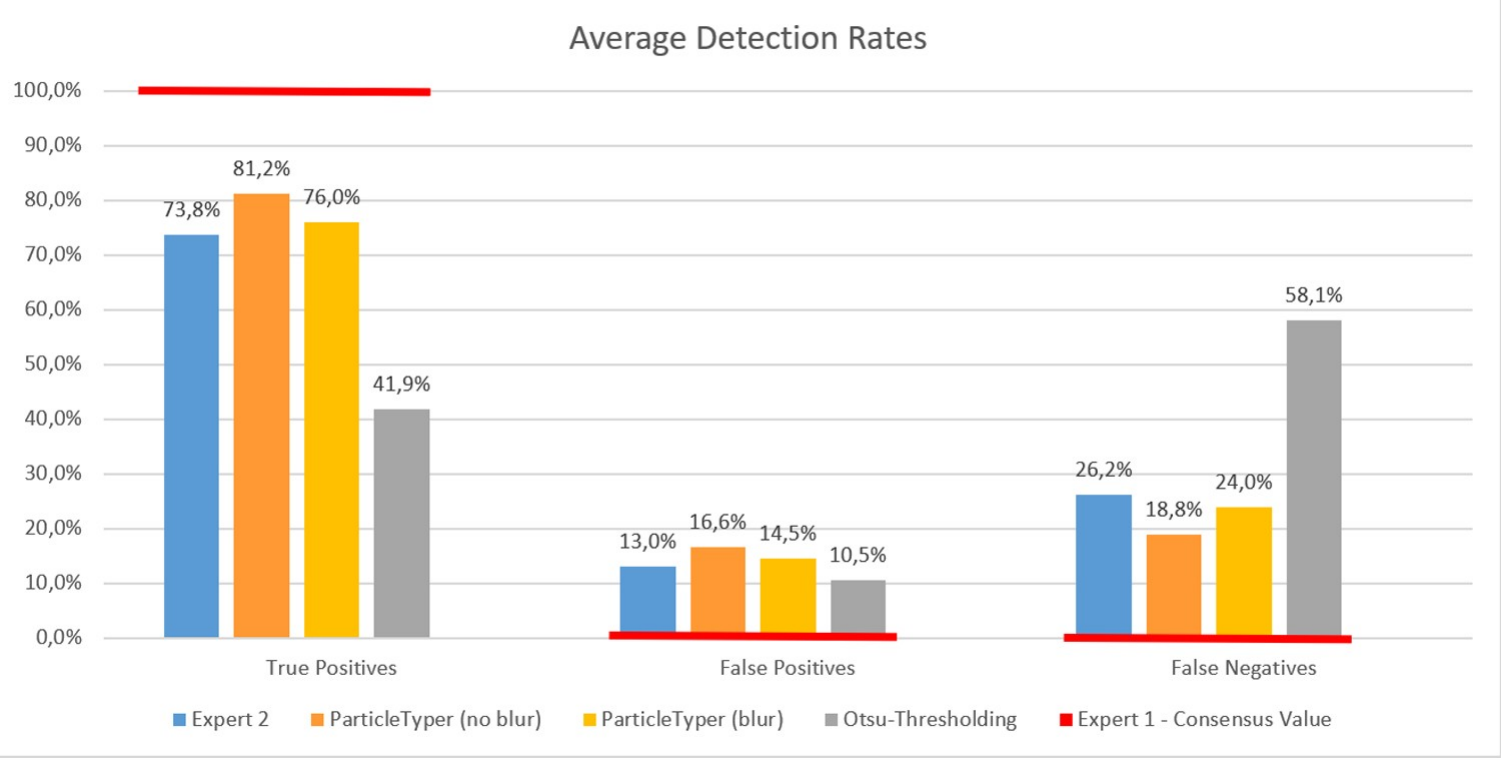
Average detection rates of second expert, *TUM-ParticleTyper* with and without blur and a program using Otsu-thresholding compared to the particles identified by expert one.

To show the program’s validity, its results were not only compared to the data created by a single expert, but also to the estimate of a second expert and the detection using *Otsu*-thresholding as in [15]. Hereby, each detected object was classified into true positive (if it corresponds to a particle also identified by the expert) or false positive (if it was not identified by the expert). Additionally, the particles identified by the expert that do not have a correspondence in the analyzed image are classified as false negative. For each classification the rate regarding the total number of particles in each test image was calculated and averaged over all seven test images to weight each test image equally. The results can be seen in Fig 9.

The *TUM-ParticleTyper* achieved detection rates that lie between the estimates of both experts for true positives and false negatives. With blurring the images, the accuracy is decreased, since the original data is altered beforehand and therefore information is lost. However, the impact is relatively minor, and it is necessary to guarantee an acceptable runtime. The software using *Otsu*-thresholding from [15] fails to detect many particles (58.1%) and is clearly outperformed. The rate of false positives for *TUM-ParticleTyper* is higher than the second expert and the *Otsu*-method. This has two reasons: First, the program is in general more sensitive than the *Otsu*-method, which detected less objects in general. It has a smaller false positive rate, since it detects only the most characteristic parts of the image, which are clearly recognizable objects. The second reason is that oftentimes objects were indeed detected in a position where also expert one marked a particle but their areas oftentimes did not fulfill the criterium “minPixels” larger than 51 in expert one’s findings but did so in the analysis with *TUM-ParticleTyper* due to small inaccuracies in the exact extraction of the contour.

For the seven SEM test images (See *TUMmedia* repository [23]), outstanding detection rates were achieved: The best parameter set achieved a detection rate of 98.3% for the true positives, accordingly 1.7% for the false negatives and only a false positive rate of 2.9%. Compared to the results from the Raman images these rates are remarkably good. However, as mentioned above analyzing too complicated SEM images may result in worse rates, due to their more complex nature.

All in all, the analysis with *TUM-ParticleTyper* generates solid results within the margin of the error of the two experts and can therefore be considered as valid alternative. The validation protocol applied here can generally be used to evaluate the performance of an image processing program.

### Method comparison of the single point approach vs. imaging

**Fig 10:**
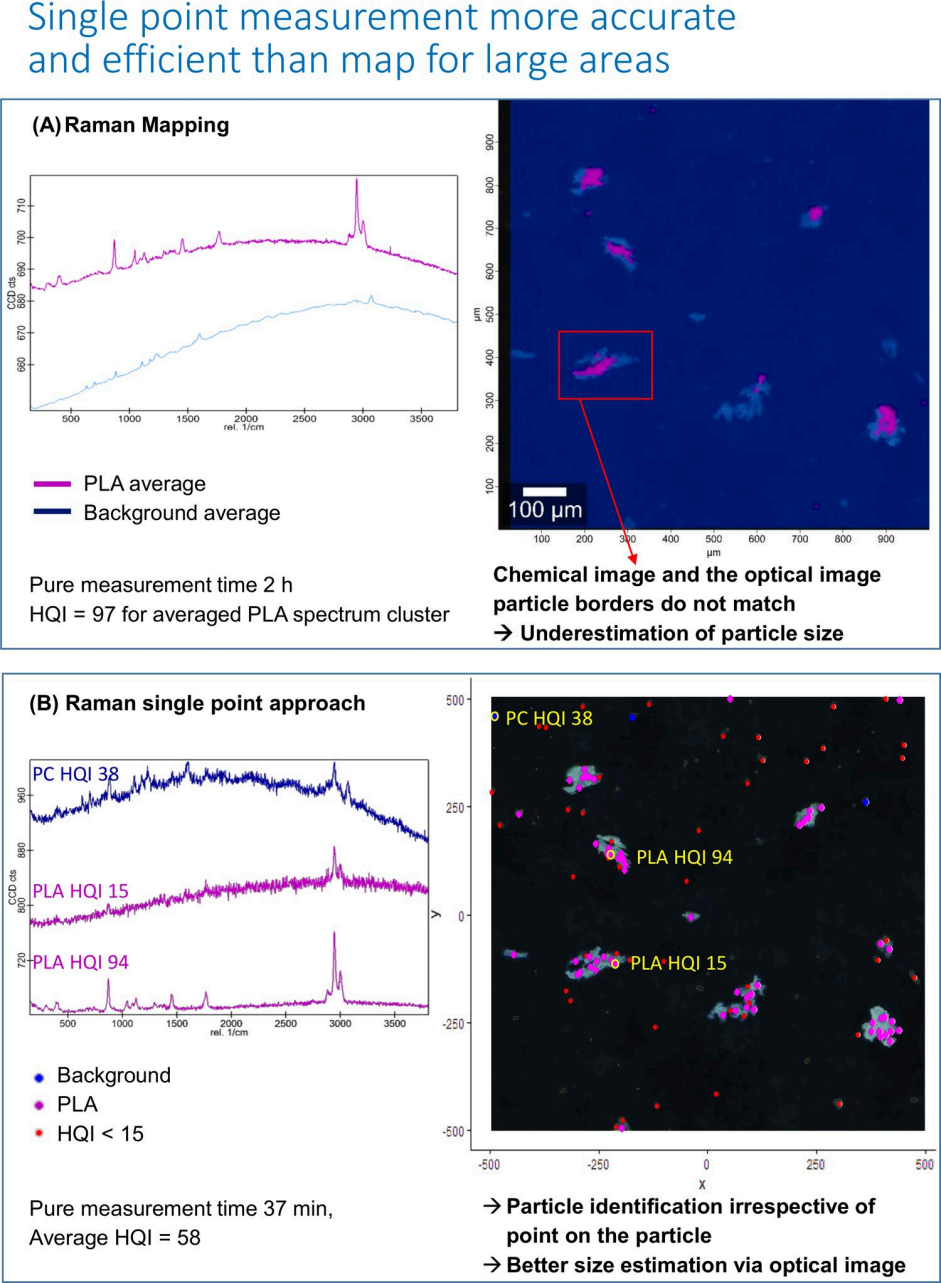
Comparison of Raman mapping vs Raman single point measurement in the same 1000 × 1000 μm square of a PLA reference sample, with the goal to analyze the area in a similar period. Mapping with 10 μm steps, 5 mW, 500ms/ scan, 532 nm laser, 20× magnification (A). Measurement of multiple points on a particle to determine if all points on a particle are equally representative, with 3 mW, 4×5s/ scan, 532 nm laser, 20× magnification (B). Purple indicates the presence of the target PLA, blue indicates the prevalence of the background polycarbonate signal from the filter. All spectra that could not be identified are marked red. For large areas, single point measurements are both efficient and representative.

An alternative to the single point measurement of particles (localization and measurement of particles at their centers) is the imaging of filter areas to analyze all particles therein, by clustering the resulting spectra and calculating the size of the particles based on the spectral signature. This approach is prominently used for the automated μ-FTIR analysis [17, 18, 26, 31] of microplastics but can also be applied for Raman microspectroscopy as demonstrated by Käppler et al. 2016 [25]. One of the drawbacks of the mapping approach is that large datasets (~ 30 GB) are created and need to be processed for spectral identification. The supposed advantage of the imaging procedure is that no particles are overlooked and that there are multiple spectra for each particle, which can be averaged to yield a clean spectrum. The single point approach on the other hand only considers one measurement position per particle, but the integration time is longer for each measurement resulting in a higher signal to noise ratio for the specific point that is measured. In our approach, a maximum of 7000 particles is analyzed resulting in a much smaller dataset and faster analysis (~315 MB for 7000 spectra). The comparison of a mapping and a single point measurement for an area of 1 mm^2^ is shown in Fig 10. In order to validate the extreme reduction of measurement points in the single particle approach, particles were localized with *TUM-ParticleTyper* and multiple measurements were performed for each particle to see if all measurement points on one particle yield equal results regardless of their position, thus proving that one point is sufficient. As can be seen in Fig 10 most measurement points yield the same spectrum for each particle. The spectra acquired within the boundaries of the particle differ solely by the achieved hit quality indices (HQI) but would have led to the identification of the particle in an average of 82% of all cases, even when the points are close to the boundary. Comparing these findings to images from μ-FT-IR imaging, it becomes clear that the signal intensity of the spectrum is highest in the particle center and decreases towards the edges [18, 31]. Furthermore, refractive errors occur for irregularly shaped materials [32] which introduces artefacts to the spectra and may lead to an underestimation of particle size, as these spectra are difficult to classify. To determine the influence of the measurement position on the HQI we correlated it to the distance of the measurement position in reference to the point, where the highest HQI was determined. The result of this analysis is that the HQI decreases when the distance to the particle center increases, which is consistent with the observations for μ-FT-IR imaging [18, 31]. When comparing our results from Raman imaging and Raman single point measurements it becomes clear that this effect will be even more pronounced when short integration times are needed for the acquisition of spectra. In our Raman images we see that less particles (7 out of 13) were identified as poly lactide and that the particle size is severely underestimated, because the spectral quality of the boundary regions is so poor that it is not classified as PLA through clustering. Specifically, the combined total area of all particles from imaging yielded 35.47 mm^2^ for seven particles (incorrectly segmented particles were joined for this count) vs. 109.3 mm^2^ for 13 particles that were chemically identified by single point measurements and morphologically characterized based on the evaluation of the optical microscopy image via *TUM-ParticleTyper*. This 82% difference in overall area could however be remedied by using a smaller step size and / or a longer integration time per scan, which would substantially increase the measurement time. With the parameters applied here a 1000 μm × 1000 μm area was measured in 2 h (3.3 times longer than the single point measurements referring to our Raman system).

We conclude that neither imaging nor single point measurement is flawless but selecting single points based on particle recognition is a valid way to reduce the overall measurement time, In addition, the morphological characterization based on image processing of the microscopy image yields better results than the size estimation based on the spectral fingerprint.

### How complex may images be to allow for successful analysis? Application to a real sample for fiber detection in washing machine water

**Fig 11:**
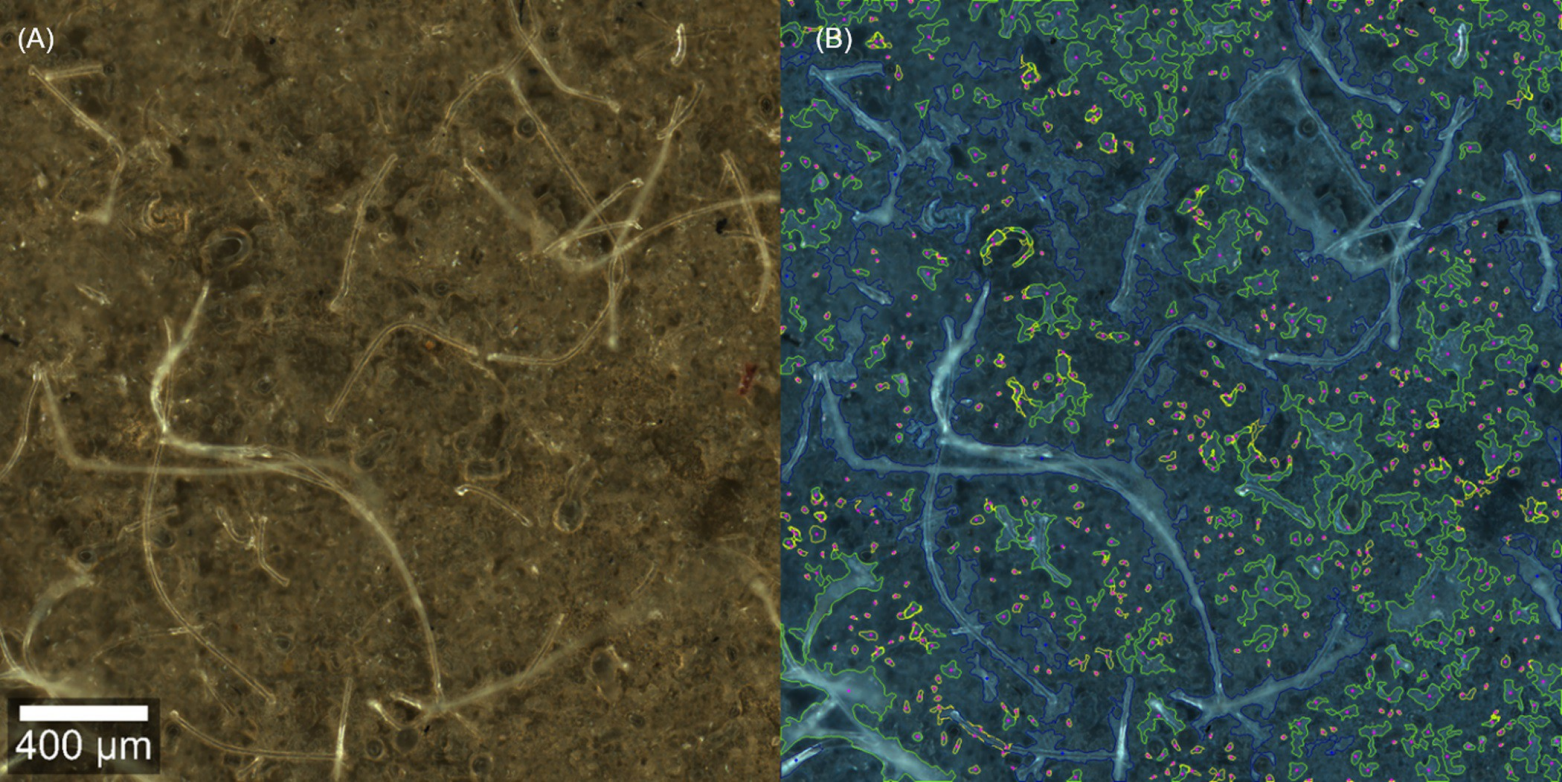
Segmentation accuracy for samples with intense matrix background. Original image (A), processed image (B). Fibers are marked in blue, particles are marked in green, but were ignored for the chemical identification, as here the focus was on fibers.

To prove that *TUM-ParticleTyper* is also able to handle very complex images, microplastic analysis was conducted in a sample of washing machine water. The aim was to detect fibers originating from synthetic clothing treated in the washing step. After the localization and morphological characterization via *TUM-ParticleTyper* 2000 of 4000 found fibers were analyzed. Thereof 320 could be automatically assigned via *TrueMatch*, and additional 109 fibers could be identified via manual assignment. The segmentation of the particles is shown in Fig 11. Due to matrix interference from dried detergent on the filter surface it is difficult to manually locate fibers. In the processed image the particle counts may not be reliable anymore, as there are too many particles on the filter surface and they are therefore detected as aggregates. The fibers on the other hand can still be localized. This shows that also complex samples can be morphologically analyzed via *TUM-ParticleTyper*. However, since the success of the particle detection critically depends on the quality of sample treatment and of the image, it is recommended to validate the performance for each sample type, image acquisition setup and research question.

### Challenges in the analysis

Despite the program’s successful performance, a few challenges remain to be resolved in further improvements. The first are holes in contours. As mentioned earlier, the program has an algorithm to move the center away from such holes to the inside of a particle (e.g. for torus-shaped particles). Nevertheless, the hole affects the calculated area of the particle, since the hole’s area cannot be calculated and subtracted easily.

A second challenge are long fibers that shape a ring. The contour might contain a huge area that is not part of the fiber, but which is nonetheless considered in the calculation of the area. Here Primpke et al. 2019 proposed the determination of fiber sizes using a skeletonize function which is superior to our fiber size estimation [33].

A third issue is the detection of agglomerated particles. Since the algorithm for contour detection cannot separate agglomerates, particles that overlap or adjoin to each other are detected as one contour and therefore as one particle. Usually an approach using a watershed algorithm allows the separation of agglomerates, but the images also contain fibers. Watershed has a poor performance on fibers and separates them into several small fractions [15]. It is therefore not suitable and not implemented so that agglomerates remain a restriction in the program.

As fourth aspect, the *TUM-ParticleTyper* can have a weak performance when the minimum area is chosen too small. Even though blurring usually removes noise, false positive detections still occur more frequently for smaller minimum sizes. Finally, SEM images can contain bright and dark objects, but only the performance on images with only bright ones can be regarded as satisfactory. An approach to overcome this challenge is the inversion of the colors of the image. Dark objects then appear as bright objects and can be detected in a second analysis.

A general problem is the fact that the program’s performance can only be validated in relative terms. There are no images with perfectly extracted particles available that would provide a defined true value. The only ways to assess the performance is to manually evaluate the image and assign a consensus value, considering that even the particle detection by two experts does not yield the same result. If the program’s output is within an acceptable range of deviation from this consensus value, we can consider it as functioning properly.

### Conclusion and outlook

*TUM-ParticleTyper* is an open access image processing tool for the morphological characterization of particles in optical, fluorescence and scanning electron microscopy images. It is the first such tool that can be calibrated to fit the camera system of the user, the requirements of the analysis, as well as the complexity of the sample. The essential part of the work presented here was not only the development of such a tool but also the development of validation protocols for the particle localization with *TUM-ParticleTyper* and the sample reduction from full filter imaging to single point measurements at the particle centres. It is recommended to prepare a test sample, to analyse it with the *TUM-ParticleTyper* and to parallelly do a manual particle identification, by marking all particles in red. The found particle number, mean area and Feret’s diameters should be compared to get a rough quality assessment, but it is important to access the true positives, false positives and false negatives as described in the protocol presented here to access the accuracy. As demonstrated a 100% accuracy is not possible to achieve with complex samples as even the assessment of two experts deviates by ~30%, which is why no ground truth can be found for the assessment only a consensus value. The protocol can be transferred to alternative systems and programs for quality control, enabling users to check their current or future analysis protocols. To enable such an analysis, the sample surface must be as flat as possible. Therefore, a filter holder was developed, produced and characterized. With the setup brought forward here, we advance Raman microspectroscopy analysis of microplastic particles to accomplish a routine, size-resolved chemical quantification of particles down to a size limit of 10 μm. Further efforts will need to concentrate on pushing this boundary towards the detection of even smaller particles.

## Supporting information

S1 File (PDF)
