# Supplementary material for: TUM-ParticleTyper: A detection and quantification tool for automated analysis of (Microplastic) particles and fibers
Source: PLoS One. 2020 Jun 23;15(6):e0234766. doi: 10.1371/journal.pone.0234766 (PMC7310837; doi:10.1371/journal.pone.0234766)
Supplement: S1 File — (PDF) [file pone.0234766.s001.pdf]

1 Supplements to the Material and Methods

1.1 Roughness testing for the development of filter holders

The suitability of the filter holder for the analysis of MP by means of RM was evaluated by measuring the surface roughness. Therefore, a gold-coated polycarbonate filter (diameters 25 mm and 50 mm, pore size 0.8 µm, Analytische Produktions-, Steuerungs- und Controllgeräte GmbH, Germany) was used. The roughness was evaluated by using clean and used filters. The used filters were in contact with an artificial matrix which was a suspension of humic acids (native, Carl Roth GmbH + Co. KG, Germany) and bentonite (Carl Roth GmbH + Co. KG, Germany) in ultrapure water (Milli-Q® Reference, Merck KGaA, Germany), which was filtered onto the filter (vacuum filtration, 25 mm, 30 mL, with glass frit, Sartorius Lab Instruments GmbH & Co. KG, Germany) and afterwards the filter was freed from the residue with ultrapure water (Milli-Q® Reference, Merck KGaA, Germany). The dried filter was clamped into the filter holder and analyzed with *TrueSurface* (WITec GmbH, DE). For comparison the same filters were laid onto a glass slide and fixed to a glass slide with a double sided tape (tesa® Doppelband TRANSPARENT, tesa SE, DE) and equally analyzed with TrueSurface (WITec GmbH, Germany) which measures the surface topography.

The roughness for filters with 50 mm diameter was evaluated on an area of 30 mm × 30 mm and a size of 100 Pixel × 100 Pixel. For filters with 25 mm diameter an area of 12 mm × 12 mm and a size of 40 Pixel × 40 Pixel was evaluated. This resulted in an equal resolution of 300 µm Pixel^{-1}. The roughness was evaluated by the maximum peak-peak distance which is the distance of the highest to the lowest pixel. The smaller this distance is, the smoother the surface and the better the fixation method.

Furthermore, images from publications were extracted and analyzed to show the generalizability of our approach. For the acquisition parameters we refer to the original publications.

1.3 Acquisition of chemical information via Raman microspectroscopy

Single point measurements:

The particles were localized (calculation of centers for Raman measurement) and morphologically analyzed (Feret's diameter min and max, area, ratio of Feret's diameter and percentage of area covered by particle in Feret's box for shape analysis) via image processing using *TUM-ParticleTyper*. Subsequent Raman microspectroscopy revealed the identity after an automated spectral assignment. Measurement parameters: 532 nm laser, 3 mW using *TruePower*, 20 s measurement time, 20× objective, inserting the determined coordinates by *TUM-ParticleTyper* via *PointViewer* on to the *alpha300R* Raman Microscope, WITec GmbH, Germany. Spectral assignment: *2 component search via correlation coefficient [1]* in the region of 600 – 1800 cm^{-1} up to a hit quality index of 15, using *TrueMatch*, Witec, Germany *see validation of these parameters in von der Esch et al. 2020[2]*.

Imaging:

The Raman imaging experiments were performed according to the procedure by Käppler et al. 2016. [3] (Measurement parameters: 1000 µm × 1000 µm area, a 10 µm step size 5 mW 500 ms/scan 532 nm laser 20× magnification on an *alpha300R* Raman Microscope WITtec GmbH, Germany). The identification of the particles is done by k-means clustering of the acquired spectra. (k=20, yielded best results using *Project FIVE*, Witec, Germany) Only the target Cluster (of the searched polymer polylactic acid) was used to calculate the number and size of the respective particles and for the overlay with the original image.

For the comparison of single point measurements and imaging the parameters were tweaked so that the same area was analyzed roughly in the same amount of time, without sample size reduction in the single point approach (no random sampling). The exact parameters for each comparison are stated next to the overlay images.

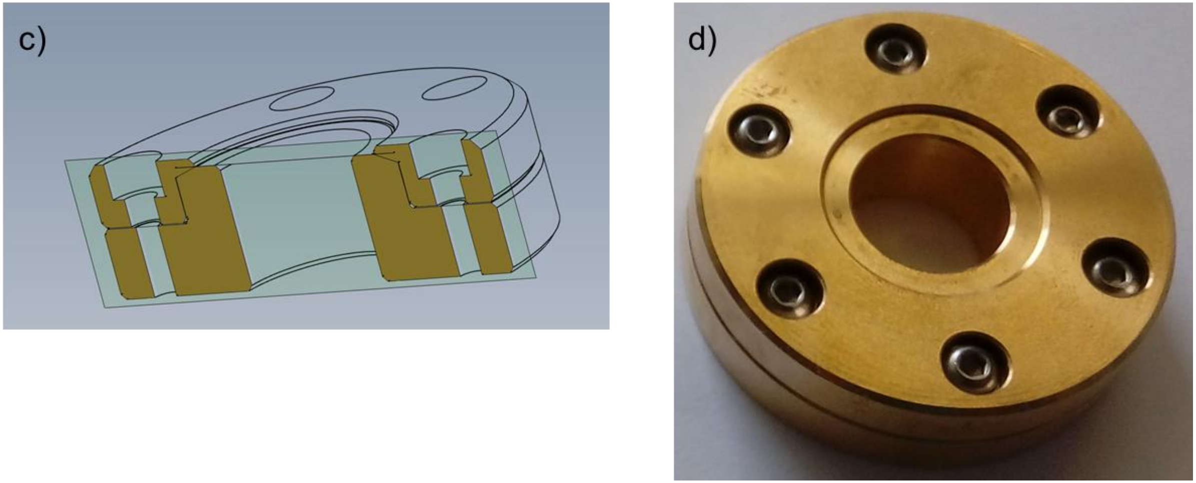

Figure 1: Exploded view of the filter holder a), massive filter holder for filters with diameters of around 25 mm b), schematic drawing of the filter holder c), filter holder with opening in the center for filters with diameters of around 25 mm d), constructed at the Institute of Hydrochemistry, Chair of Analytical Chemistry and Water Chemistry, TUM.

Two sizes for filters with 50 mm diameter and for filters with around 25 mm (Figure 1) and two types of filter holders were constructed. The first type is a massive one (Figure 1b), the second type possesses an opening in the center that can be used for transmission measurements or illumination from the downside of the filter (Figure 1).

For fixation of a filter the upper part of the filter holder is lifted. The filter is placed onto the lower part and the upper part is laid down on the filter carefully. By tightening the screws in an alternating manner, the filter is smoothly fixated between the two parts of the filter holder.

In Table 1 the peak-peak distances for clean gold-coated polycarbonate filters with 50 mm and 25 mm diameter as well as the peak-peak distances for the same filters that were previously in contact with an artificial matrix are given. For the calculation of the mean and the standard

Laid on glass slide	99.2 µm ± 2.6 µm
Gold-coated polycarbonate filter with 50 mm diameter; contact with artificial matrix	
Filter holder with opening in the center	34.8 µm ± 5.0 µm
Glued to glass slide	466.3 µm ± 1.7 µm
Laid on glass slide	871.4 µm ± 3.0 µm
Clean gold-coated polycarbonate filter with 25 mm diameter	
Massive filter holder*	5.8 µm ± 2.1 µm
Filter holder with opening in the center*	10.4 µm ± 2.3 µm
Glued to glass slide	121.4 µm ± 2.5 µm
Laid on glass slide	63.1 µm ± 11.8 µm

al. Analysis of environmental microplastics by vibrational microspectroscopy: FTIR, Raman or both? Anal Bioanal Chem. 2016;408(29):8377-91.
